# Primary small cell carcinoma of the pancreas: rare type of pancreatic cancer and review of the literatures

**DOI:** 10.1186/1477-7819-10-32

**Published:** 2012-02-08

**Authors:** Dansong Wang, Yefei Rong, Wenchuan Wu, Dayong Jin

**Affiliations:** 1 Pancreatic cancer group, Department of General Surgery, Zhongshan Hospital, Fudan University, Shanghai, 200032, China

**Keywords:** Small cell carcinoma, pancreatic neoplasm, diagnosis, therapy

## Abstract

**Back ground:**

Primary small cell carcinoma of the pancreas (SCCP) is a rare malignancy with an extremely poor prognosis which accounts for 1 to 1.4 percent of all pancreatic malignancies.

**Case presentation:**

We present the case of a 62-year-old man with a half-month history of upper abdominal discomfort who was diagnosed with SCC of the pancreatic tail. A Chest X-ray showed no evidence of primary lung tumor. The diagnosis of a SCCP was confirmed by post-surgery pathology and immunohistology. In our review of the published reports of SCCP, we only found a few cases reported in the literatures. The diagnosis of SCCP needs the post-surgery pathology and immunohistology and the prognosis of SCCP is extremely poor. There was a significant increase in median survival, from 1 to 6 months, in treated patients compared to patients treated only by symptomatic management. Chemotherapy was the most common treatment and the combination of cisplatin/etoposide was most frequently prescribed.

**Conclusion:**

The accurate diagnosis of (SCCP) is necessary for determining prognosis and deciding appropriate therapy.

## Background

Small cell carcinomas (SCCs) is an aggressive tumor, which account for 18-20% of all primary lung cancers, but they are also described in the urinary bladder, prostate, salivary glands, pharynx, larynx, esophagus, stomach, pancreas, colon, rectum, skin, and cervix [1-3]. Similar to SCC of the lung, SCC of the pancreas (SCCP) is a lethal disease and its prognosis is extremely poor. If untreated, it progresses rapidly with fatal course. We hereby reported a case of SCCP and reviewed the articles associated with the SCCP.

## Case presentation

**Figure 1:**
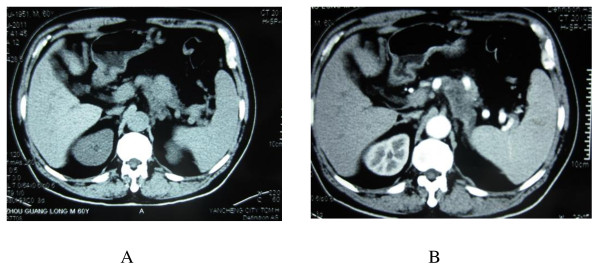
**Computed tomography (CT) scan of the upper abdomen**. There was a homogeneous mass in the pancreatic body and tail by noncontrast CT scan (A). In the arterial phase the mass was in low density (B).

The patient was a 62-year-old man. Except hypertension, he had no significant past medical history. He went to our hospital on July 21, 2011, with the symptom of upper abdominal discomfort. The local hospital CT scan revealed a low-density mass at the pancreatic tail (Figure 1). Laboratory examination: Hemoglobin: 149 g/L; white blood cell count: 4.7 × 10^9^/L; platelets: 78 × 10^9^/L; AST: 21 U/L; ALT: 14 U/L; total bilirubin: 14.2 μmol/L; direct bilirubin: 6.6 μmol/L; serum creatinine: 75 μmol/L;AFP:2.2 ng/mL (normal < 20 ng/ml); CA19-9:122.7 U/mL (normal < 37 U/mL); CEA: 2.82 ng/ml (normal < 5 ng/mL). Chest X-ray had no sign of primary lung cancer and metastasizes. In addition, there was no evidence of ascites, and liver metastasizes; therefore exploratory laparotomy was performed routinely.

**Figure 2:**
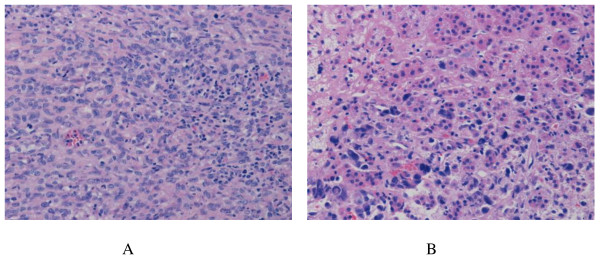
**Histology of the SCCP**. HE stains demonstrated the small cell character of the SCCP. There are nests of small to medium-sized cells with scanty cytoplasm and hyperchromatic nuclei (A, ×200). Metastasis was found in the adrenal gland (B, ×200).

**Figure 3:**
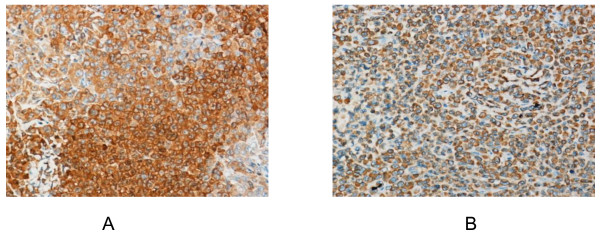
**Immunohistochemistry of the SCCP**. The tumor cells were positive for NSE (× 200, Figure 3A) and Chromogranin A (× 200, Figure 3B).

At abdominal exploration, a 6 cm mass was found in the pancreatic tail. So we performed distal pancreatectomy with splenectomy. All pathology specimens were routinely processed. Histological examination showed spindle-shaped cells with scanty cytoplasm and hyperchromatic nuclei (Figure 2A). Metastasis cells were found in the left adrenal (Figure 2B). Immunohistochemical stains were performed on the paraffin-embedded sections. The tumor cells demonstrated positive reaction to neuron-specific enolase (NSE) and chromogranin A (Figure 3). The patient was discharged on postoperative day15 in stable condition. The patient is followed up and currently performing chemotherapy.

## Discussion

SCCP is a rare neoplasm, with only a few cases reported in the literatures (Table 1). Among all the primary pancreatic neoplasms, only 1% neoplasms are SCCP [4]. Preoperatively, it is difficult to distinguish SCCP from pancreatic adenocarcinoma by imaging studies. However, some studies have indicated that imaging studies might be helpful in the differential diagnosis of SCCs of the pancreas [5,6]. In 2000 Ichikawa et al. [5] reported three cases of SCCP. In each case, the tumor arose from the head of the pancreas as a large (size 5-7 cm), homogeneous mass by noncontrast CT. Furthermore, all the tumors showed minimal contrast after intravenous contrast injection. Although pulmonary SCC was frequently associated with paraendocrine syndromes; extrapulmonary SCC does not accompanied with identifiable paraneoplastic syndromes frequently. Only two reported that SCCP had elevated hormones levels: one with adrenocorticotrophic hormone (ACTH) secretion [7] and the other with paraneoplasic hypercalcemia [8]. Neuron-specific enolase (NSE), which is found in neuroendocrine cells, is a good marker for the diagnosis of pulmonary SCC [9,10]. Furthermore, Johnson et al. [10] reported that serum NSE concentrations correlated with the extent of the disease and response to treatment. In other reported cases, NSE was increased also in SCCP [11,12]. Therefore NSE could be considered as a tumor marker and could be used for diagnosis or assessment of treatment effect in patients with SCCP [12,13]. In our case, we checked the serum NSE post-surgery (20.9 ng/ml) which was still above normal level (< 15.2 ng/ml). Nakamura et al [12] reported that serum carcinoembryonic antigen (CEA) concentrations might also be used for assessment of treatment effect but not good for the differential diagnosis, because the presence of a high CEA concentration in patients with SCCP is variable (in our case, the serum CEA was normal) and CEA is not specific for SCCP but also frequently increases in patient with pancreatic ductal adenocarcinoma. Pro-GRP is known to be more stable than GRP and is more sensitive and specific tumor marker than NSE in pulmonary SCC [14] and also in extrapulmonary SCC [15]. Matsubayashi et al [11] suggests that Pro-GRP could also be an important marker for diagnosis and assessment of treatment in patients with SCCP.

**Table 1:** Summary of the clinical data reported cases of small cell carcinoma of the pancreas

**Ref**.	Age	Sex	Location	Treatment	Survival
4	42	M	Body	Symptomatic	1 month
4	67	M	Head	Symptomatic	1 month
4	62	M	Head	Symptomatic	2 months
4	54	M	Head	Symptomatic	2 months
4	73	M	Head	Symptomatic	1 month
5	45	M	Head	Symptomatic	Not stated
5	75	M	Head	Symptomatic	Not stated
6	27-60	3F/3M	Head	Surgery and Chemoradiotherapy	9-173 months
7	50	F	Tail	Symptomatic	2 weeks
8	66	M	Head	Symptomatic, polychemotherapy followed by CDDP VP-16	2 week
12	69	F	Head	Surgery and CDDP, VP-16	6 months
13	62	M	Head	CDDP, Doxorubicin, VP-16	2 month
18	54	M	Head	Polychemotherapy followed by CDDP, VP-16	50 month
19	64	F	Not stated	Carboplatin, etoposide	56 months
19	69	M	Not stated	Carboplatin, etoposide	14 months
19	62	M	Not stated	Carboplatin, etoposide	9 months
19	69	M	Not stated	Carboplatin, etoposide	18 months
20	74	M	Tail	Gemcitabin, PEGylated octreotide, 5-FU	8 months

CDDP: cis-dichlorodiammine platinum; VP-16: etoposide; 5-FU: 5-fluorouracil

In a review of all published cases of SCCP, 91% have metastasizes at the time of initial diagnosis. In Bertrand et al report, the most frequent sites of metastasis are the peripancreatic lymph nodes (62%), the liver (38%), the lungs (14%), the bone marrow (14%), the bone (10%), the colon (10%), and the adrenal gland (10%); rarer sites included the spleen, gallbladder, kidney, skin and brain [16]. In our case, the SCCP had metastasizes in the left adrenal gland. Therefore the majority of patients had no opportunity for their tumor-resection. Until now only few cases had the tumor-resection. Winter et al [6] reported 6 cases SCCP who had the surgery. These patients were followed with adjuvant chemoradiotherapy. Three of six patients survived more than 2 years, and two patients survived over five years. The patient who lived for 173 months represents the longest reported survival for pancreatic SCC to date. The median survival was 20 months, which is comparable to patients with resected ductal adenocarcinoma of the pancreas [17]. Their research makes us believe that combined surgery with adjuvant chemoradiotherapy might improve the prognosis of SCCP.

As for the pulmonary SCC, the encouraging long remission rates could be achieved by chemotherapy and/or radiation. Surgery alone is generally unsuccessful in managing either pulmonary or extrapulmonary SCC [16]. Because the combination of cisplatin/etoposide is most frequently prescribed in SCCP, this combination is also widely used in SCCP. In 1989, Morant et al [18] reported complete remission of refractory SCCP with cisplatin and etoposide. Initial chemotherapy with streptozotocin, 5-fluorouracil, and methotrexate, doxorubicin, cyclophosphamide, and lomustine (MACC) had been unsuccessful. They used a schedule consisting of etoposide and cisplatin which made the patient remain in complete remission 50 months after the diagnosis. Sakamoto et al [19] diagnosed 4 cases of SCCP with endoscopic ultrasonography-guided fine-needle aspiration (EUS-FNA). All patients were treated with combination chemotherapy using a schedule consisting of carboplatin and etoposide. Three patients treated with the combination chemotherapy achieved remission, two with a complete response and one with a partial response. The remaining one patient showed no change. One of the two patients with a complete response survived for 56 months following the diagnosis. Recently, Berkel et al [20] demonstrated that local tumor control could be achieved with gemcitabine once a week and a long-acting somatostatin analogue once a month; however, liver metastasis in their patient showed progression. Bertrand et al [16] made a review on the SCCP. In their report, patients receiving either systemic or local therapy showed a significantly higher median survival compared to patients treated with symptomatic management alone (6 vs. 1 month, P ≤ 0.0001). However, the authors found no significant difference in median survival between patients receiving chemotherapy alone and patients given local treatment in addition to chemotherapy, although it should be noted that the latter group contained only three patients. Overall, median survival was just 3 months (range 0.5-50 months). Therefore, a standard treatment for unresectable SCC of the pancreas has not been established at yet. Recently, Yachida et al [21] established a new cell line (A99) from a primary SCCP patient. A99 cells were positive for chromogranin A, NSE. The establishment of this cell line may help in studying the cell biology of SCCP or for evaluating novel targeted agents in preclinical models.

## Conclusions

The SCCP is a rare type pancreatic cancer and has poor prognosis; therefore the accurate diagnosis of SCCP is necessary. Whether the patients had their tumors resected, the adjuvant chemotherapy should be performed, and cisplatin/etoposide appears to be the preferred chemotherapy regimen at present.

## Consent

Written informed consent was obtained from the patients for publication of this report. A copy of the written consent is available for review with the Editor-in-Chief of this journal.

## Competing interests

The authors declare that they have no competing interests.

## Authors' contributions

DSW, YFR and DYJ: Operating team and drafted the manuscript; WCW: operating team, post-surgery care. All authors read and approve the final manuscript.
